# Artificial Intelligence Can Improve Patient Management at the Time of a Pandemic: The Role of Voice Technology

**DOI:** 10.2196/22959

**Published:** 2021-05-25

**Authors:** Tomasz Jadczyk, Wojciech Wojakowski, Michal Tendera, Timothy D Henry, Gregory Egnaczyk, Satya Shreenivas

**Affiliations:** 1 Department of Cardiology and Structural Heart Diseases Medical University of Silesia Katowice Poland; 2 Interventional Cardiac Electrophysiology Group International Clinical Research Center, St. Anne's University Hospital Brno Brno Czech Republic; 3 The Carl and Edyth Lindner Center for Research and Education The Christ Hospital Cincinnati, OH United States

**Keywords:** artificial intelligence, conversational agent, COVID-19, virtual care, voice assistant, voice chatbot

## Abstract

Artificial intelligence–driven voice technology deployed on mobile phones and smart speakers has the potential to improve patient management and organizational workflow. Voice chatbots have been already implemented in health care–leveraging innovative telehealth solutions during the COVID-19 pandemic. They allow for automatic acute care triaging and chronic disease management, including remote monitoring, preventive care, patient intake, and referral assistance. This paper focuses on the current clinical needs and applications of artificial intelligence–driven voice chatbots to drive operational effectiveness and improve patient experience and outcomes.

## Review of Voice Assistants in Health Care

A cutting-edge development in the field of artificial intelligence (AI) and machine learning has enabled verbal communication interfaces between users and voice assistants (VAs), which are synonymously termed “voice chatbots” or “conversational agents.” VAs, exemplified by Apple’s Siri, Amazon Alexa, and Google Assistant, are a software layer embedded in a standalone smart speaker, such as Home Pod, Amazon Echo, Google Home, or smartphones, allowing for the interpretation of human speech. Technically, conversational agents are cloud-based services performing speech-to-text and text-to-speech tasks that are initiated by a person evoking a wake-word followed by a voice command [1]. The implementation of VAs in health care has the potential to support the delivery of care in a routine clinical setting, especially when functionalized by a computerized clinical decision support system (CDSS). These robotic process automation (RPA) chatbots have the decision-making ability of a human health care professional to perform rule-based tasks (ie, digital patient triaging) [2]. Voice technology has already been tested in various applications to support everyday clinical activities [3-12]. Its applications include the following:

1. Education-level services answering frequently asked questions based on a knowledge database (eg, first aid instructions).
2. Optimization of processes (eg, medication reminders, prescription refills, appointment scheduling, bedside assistants, and paperless documentation).
3. Patient support with personalized rule-based clinical instructions (eg, instructions to reduce carbohydrate intake among patients with diabetes mellitus).
4. Data collection services (eg, collection of patient-reported outcomes, biometric tracking, and identification of health status changes exemplified by the collection of medical history or remote home monitoring; classified by the Food and Drug Administration [FDA] as Medical Device Data Systems [13,14]).
5. Medical device–grade solutions designed to diagnose, treat, cure, mitigate, or prevent disease, termed “Software as a Medical Device” by the FDA [15], combining voice interface and CDSS.

Education-level VA services have been implemented by health care organizations and provide guidelines, instructions, and navigation for patients, such as WebMD [16], Cleveland Clinic’s Tip of the Day [17], Boston Children’s KidsMD [18], Mayo Clinic’s First Aid [19], American Red Cross’ First Aid [20], Mayo Clinic’s News Network [21], Boston Children’s My Children’s Enhanced Recovery After Surgery [22], Ohio Health [23], or New Hanover Regional Medical Center [24]. For example, Mayo Clinic’s First Aid application deployed on Amazon Alexa provides self-care instructions for everyday mishaps. Through a voice interface, users can ask for guidance on how to treat fever or what to do in case of spider bites or burns. These examples confirm the applicability of conversational systems for responding to health-related search activities. Moreover, the Livongo Blood Sugar Lookup app is designed for patients with diabetes mellitus, which supports them to keep track of their blood sugar levels logged via Livongo meter [25]. This solution provides recent blood glucose readings through verbal input.

The optimization of processes and workflow with VAs has proved useful for patients in the pharmacotherapy management at home. The Alexa-based Giant Eagle Pharmacy app allows users to set reminders and helps refill prescriptions through home delivery services [26]. Similar functionality is available through the Express Scripts app [27]. Furthermore, Swedish Health Connect by Providence St Joseph Health allows for scheduling of medical visits by suggesting the next available appointments near the patients’ homes [28]. Similarly, the Atrium Health app provides information about the nearest urgent care service and hospital wait times, hours of operation, and contact details, thus allowing patients to schedule a same-day visit with a care provider [29]. OrbitaASSIST voice technology was also implemented as a bedside assistant to optimize communication with care teams and smart routing of requests [30]. Moreover, researchers from the Nationwide’s Children Hospital (Columbus, Ohio) designed the SpeakHealth voice interactive service for the care coordination of children with complex medical problems [3], which confirms the applicability of voice-enabled technology in pediatrics. Conversational agents have been also shown to optimize hospital operations at Cedars-Sinai Medical Center (Los Angeles, California), supporting health care professionals in time-consuming paperwork tasks and automating medical data collection and documentation through the electronic health record (EHR)–integrated CardioCube voice app (accuracy=97.5%) [7].

Personalized clinical instructions are an important component of a holistic medical approach allowing for the continuity and coordination of care. Answers by Cigna tracks patient incentive programs, provides wellness tips, and enables health coach programs to navigate treatment plans [31]. OrbitaCONNECT provides a virtual health assistant for chronic, pre-, and postvisit care continuity [32]. Furthermore, with Talkspace Alexa skill, users can access mental health assessments and a library of mental health tools [33].

**Figure 1:**
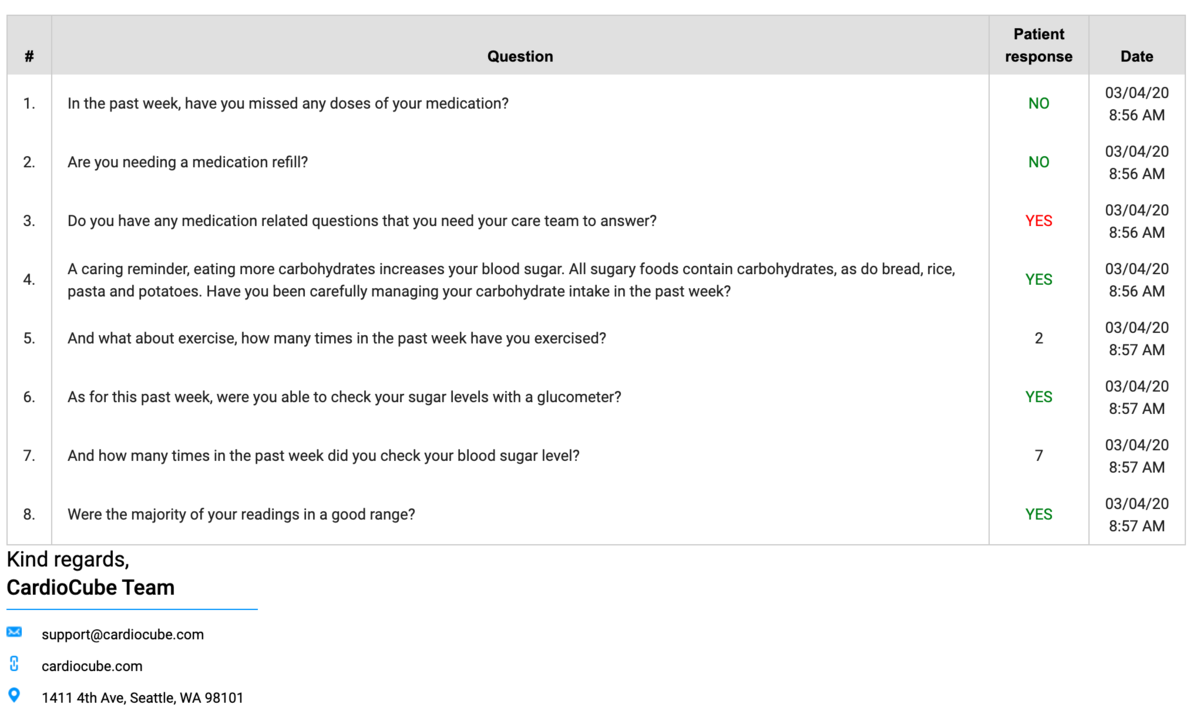
Medical report generated automatically from the artificial intelligence–driven CardioCube voice app for patients with diabetes.

End-to-end solutions powered by voice AI can be used for routine clinical care. CardioCube allows for the collection of patient-reported outcomes and biometric data captured at patients’ homes. At the appointed time, the voice assistant has a medical conversation with the patient by asking him/her, “What’s your blood pressure?” Accordingly, the patient measures his/her blood pressure by using a standard monitor and reads out the results to the voice assistant. CardioCube also asks about dyspnea, quality of life, or prompts for tasks including, “Let’s check your ischemic and bleeding risk again,” in reference to CHA_2_DS_2_-VASc/HAS-BLED scores. Afterward, CardioCube automatically transmits the results to a proprietary server integrated with the EHR system and red-flags any alarming reports. This solution, which complies with the Health Insurance Portability and Accountability Act of 1996 (HIPAA) and the General Data Protection Regulation, was validated at Cedars Sinai Medical Center [7] and classified by the FDA as a Medical Device Data System. CardioCube was also implemented for remote home monitoring of adult patients with heart failure and diabetes at the Family Care Network (Bellingham, Washington) (Multimedia Appendix 1 and Figure 1 exemplify the utility of the CardioCube voice app for patients with diabetes mellitus and a medical report automatically generated from the conversation with the virtual assistant, respectively).

Furthermore, focusing on cardiovascular diseases, Shara et al [34] at Medstar Health Research Institute conducted a clinical trial using Amazon Alexa as an automated personal health care assistant for patients with heart failure to optimize clinical care (trial registration# NCT03707275). Outcome measures included the change in the number of hospitalizations and medication adherence during a 3-month follow-up period (upon study completion, when awaiting for publication of the results). VAs have been also implemented in other fields of medicine. For example, Beaman et al [5] at the Oklahoma State University Center for Health Sciences initiated a study to examine whether verbal responses collected through Amazon Alexa is effective in capturing participant depression levels, using Patient Health Questionnaire-9 (trial registration# NCT04609267) [5].

The most advanced medical software solutions are defined to be SaMD, which, under the Federal Food, Drug, and Cosmetic Act include services designed to diagnose, treat, cure, mitigate, or prevent disease. Interestingly, regulatory authorities tend to facilitate the implementation of chatbot technology into clinical practice, especially in the context of the current pandemic. For example, the Danish COVID-19 RPA triaging chatbot that incorporates if-then branching logic was evaluated as being of “lower risk” by the FDA despite featuring a diagnostic component when the Federal Food, Drug, and Cosmetic Act was not enforced [35]. To our knowledge, none of the current VA-deployed health care apps are classified as SaMD.

## Gaps in Health Care Delivery Exposed by the COVID-19 Pandemic

The surge in COVID-19 cases has placed unprecedented strain on health care systems, requiring adjustments in treatment delivery to patients. Despite the fact that the traditional clinical approach was partially substituted with web-based visits, the mismatch between demand and resources is a realistic challenge. The capacity of health care systems to adjust is limited by the incremental rate at which systems can grow by training new health care providers and reorganization of the structure [36]. However, the exponential increase in COVID-19 cases has highlighted gaps in the current organizational systems at a global scale [37]. As a result of the number of COVID-19 cases, health care providers have been required to work on the front line, shifting the human resources available for routine care services. Clinical institutions underwent extraordinary reorganizations to accommodate for the surge in COVID-19 cases. The focus was on emergency care, scaling up beds in intensive care units, and reassigning roles among the clinical staff. Parallelly, nonurgent procedures and elective surgeries were postponed. Furthermore, shortages of personal protective equipment, especially in the early phase of the pandemic, exposed health care workers to the infection and the incapability to combat the COVID-19 pandemic [38-40]. Subsequently, we observed an unprecedented health care crisis having a direct and indirect impact on medical patients with and those without COVID-19. Combined with a high number of deaths and serious illness among patients with COVID-19 [41], there were reductions in accident, emergency, and hospital admissions for urgent conditions, such as myocardial infarctions [42], which reflects patients who decided not to seek medical care owing to the fear of becoming infected. Disruptions due to the pandemic affected people with chronic conditions who could not access routine medical services. It let to postponed elective procedures, on-site visits, and reduced rates of hospitalization during the COVID-19 emergency. This will likely result in an extensive workload in the postpandemic period.

The health care crisis magnified the problem among socioeconomic statuses and racial groups [43]. Furthermore, as pointed out by the World Health Organization, public health gaps impacted the security and economic situation [44], thus revealing deep underlying problems in the insurance coverage system in the United States. A sudden wave of unemployment caused many people to lose employer-sponsored insurance coverage, thus limiting access to care in low-income populations. The COVID-19 pandemic also impacted employers who faced several challenges when operating in a difficult organizational situation. Some institutions including hospitals were required to screen all employees and visitors for COVID-19 symptoms prior to entrance. For example, University of California San Francisco Health was posed with a tremendous logistical challenge, which was solved by using a chatbot technology [45].

## How Can Voice Technology Fill the Gaps?

The inability to provide in-person clinical consultations owing to the COVID-19 pandemic has fast-tracked the implementation of telehealth services. The use of virtual care solutions increased up to 10-fold within a few weeks, thus enabling patients to access clinical care remotely [46], with the majority including real-time, synchronous communication between patients and health care providers [47]. Of note, this approach is time- and resource-intensive and is thus inefficient for large patient populations. In mature telehealth systems, one telenurse can monitor up to 250 patients remotely; however, a single patient can be contacted via the telephone only periodically [48]. The COVID-19 pandemic forced an immediate implementation of new digital technologies [49,50], particularly AI-driven medical chatbots. These chatbots have the potential to improve access to health care through acute care triaging. This is favorable for COVID-19 screening and chronic disease management (long-term follow-up at home, scheduling of medical visits, and preventive care) [51]. Numerous health care systems have already utilized interactive voice response systems and chatbots to run hotlines helping to triage patients during the COVID-19 pandemic [45,52] for organizational optimization, including Massachusetts General Hospital and Brigham and Women’s Hospital (Boston, Massachusetts; >40,000 digital encounters/week) [2,52], OSF Healthcare (Peoria, Illinois; >50,000 digital encounters) [53], and Providence (Seattle, Washington; >150,000 messages exchanged each day between the chatbot and users) [44].

With a user-friendly and accessible interface, voice AI chatbots provide a tool for prehospital triaging at the digital front door, assessing the clinical status of patients before they make direct contact with a health care provider. McGill University Health Centre is testing the application of a COVID-19 screening tool that uses Amazon Alexa to automatically survey patients visiting Cardiology Heart Failure Clinic (trial registration# NCT04508972) [54]. Apple’s Siri provides a self-assessment tool that allows users to survey for potential COVID-19 symptoms. An automatic COVID-19 triaging service, developed on Amazon’s Alexa platform, was generated by the Mayo Clinic (Rochester, Michigan) in accordance with the guidelines of the Centers for Disease Control and Prevention and fielding a substantial number of digital encounters about COVID-19 [4]. Furthermore, Apple, Amazon, and Google have removed unofficial COVID-19–related voice apps, thus preventing potential misinformation from being spread [55].

As indicated above, a mobile-responsive, web-based interface chatbot was successfully used to screen health system employees at University of California San Francisco Health, which conducted over 270,000 digital screenings within 2 months of operation. Digital solutions have optimized organizational workflow and reduced wait times for employees entering the hospital building and prevented at-risk people from coming to work [45]. OrbitaENGAGE is a voice and chat virtual assistant solution that automates critical patient engagement workflows at the so-called “digital front door” of health care. Patients interact with a voice or chatbot VA to obtain answers to health-related questions, find locations and specialists, and access symptom screening and monitoring tools for COVID-19 or other conditions including anxiety and depression [56].

Voice chatbots can potentially help patients easily communicate their health status by providing them with any disease management data. This approach allows for remote monitoring of medical patients without COVID-19 and those with COVID-19 who are mildly ill. The implementation of RPA technology integrating medical data collected through a conversational interface with the hospital database and alert-based CDSS delivers a powerful architecture that can function hand-in-hand with health care providers. Automatic clinical follow-up services provide access to up-to-date information about the individual’s health status for informed medical decision-making [57] and reduce the risk of exposure and infection during face-to-face contact. More efficient patient care may help prevent unnecessary exposures due to decreased use of personal protective equipment (as exemplified by the web-based chatbot at Massachusetts General Hospital and Brigham and Women’s Hospital) [2].

The implementation of innovative strategies based on VAs provides support to traditional telehealth approaches and may help reduce costs of health care services by lowering the entry bar for uninsured individuals. Direct-to-consumer digital health is a growing industry that can address unmet health care needs bypassing the traditional model (eg, that used by insurance companies), thus linking patients directly with services and providers without copays and deductibles [58]. Voice AI–supported virtual health care based on video consultations could be an alternative for people who lost employer-sponsored insurance during the COVID-19 pandemic. Virtual Care by CardioCube tests the aforementioned solution in Washington [59].

## Advantages of VAs

From a user’s perspective, the advancements in the field of voice-enabled technology allow for a human-like verbal communication between users and chatbots. In contrast to text-based mobile or web-based equivalents, voice chatbots have personalized speaking styles and emotions, thus providing a more natural and intuitive experience, which is an important advantage over conventional chatbots [60,61]. Exchanging a smartphone screen with a hands-free VA might remove technological barriers. Senior users were found to prefer a conversational interface to touchscreens in a smart home environment [62]. It is also important to consider patient privacy when designing these tools. Developing multimodal solutions allows the user to choose the input modality to best suit their immediate surroundings. The user may not be comfortable speaking to VAs while in the presence of others. Having the option to provide and receive data on screen would enable them to adhere to their protocols in public environments. A strong example for the acceptance of VAs is the Healthy Coping voice bot deployed on Google Home. It is specifically targeted to patients with type 2 diabetes mellitus. The majority of users (80%) selected the voice interface over mobile solutions. Healthy Coping was assessed as easy to use and physically convenient with understandable language and communication [63]. According to Slavik et al [64], audible user interfaces are promising solutions for people with special needs for information access and control. Summarizing the advantages of voice technology, Fisher presented 5 main reasons why conversational agents may emerge as the next operating system: versatile, omnipresent, innate, contextual, and efficient characteristics (summarized as “VOICE”) [65].

From a practical perspective, VAs can automate traditional telehealth services that require human providers to operate. Using conversational agents, it is possible to collect and share information at the levels of public health and individual patients. Voice chatbots can support routine care through automatic at-home monitoring, triaging, screening, providing medical recommendations and guidelines, and improving operational workflow. It is possible to help hospitals reduce their infection risk and exposure of medical staff by automatic paperless and hands-free scripting services including dictating of visit notes, charting, and patient onboarding [66].

In regard to rapid implementation, chatbot solutions are nearly off-the-shelf products that do not require substantial information technology and server infrastructure if applied with a dedicated dashboard for clinicians. The relatively low cost and rapid adoption is another important advantage of conversational agents for web-based care delivery [2].

Commercial adoption of voice technology confirms customer acceptability and provides strong grounds for the scalability and implementation of medical applications. The supportive evidence comes from the National Public Radio and Edison Research’s “Smart Audio Report,” which shows that there are 157 million voice devices in US households [67]. Moreover, Statista projected that the number of digital VAs in use will rise to 8 billion worldwide by 2023 [68].

## Risks and Challenges

Health care systems have been working on implementing telemedicine programs for a number of years [69,70]. This leads to the question of why the uptake of web-based care solutions has been limited despite their salient clinical and economical potential. Key barriers that delay the adoption of new solutions and practice patterns exist at many levels in the chain of health care delivery [71,72]. Patient-specific obstacles include difficulties interfacing with the technology and the lack of motivation to follow through with computer-derived advice and instructions. Both patients and physicians may not wish to adopt telemedicine, as it is an imperfect surrogate for building human relationships between patients and physicians. Furthermore, both physicians and payors may be resistant to invest in web-based health platforms until these platforms have been proven to improve patient outcomes and cost-effectiveness metrics.

Initial implementation of VAs showed discordance between the quality of COVID-19–related content and guidelines by public health authorities, which have caused the dissemination of imprecise information [57]. Learning from this example, it is crucial to ensure the deliverability of reliable content through close collaboration among technology providers, developers, and health care experts. Apart from the distribution of verified information, VAs processing medical data must comply with the HIPAA to protect health information reported by end-users. Importantly, the US Department of Health and Human Services, Office for Civil Rights, waived penalties for HIPAA violation against medical providers who use everyday communication technologies to help patients remotely during the COVID-19 pandemic [39]. Furthermore, the FDA announced that the Federal Food, Drug, and Cosmetic Act will not be enforced for low-risk applications developed to combat COVID-19 [57]. These regulations supported the deployment of voice technology in clinical settings [73]. Nevertheless, it is important to establish clear regulations and guidelines for the application of voice chatbots for medical purposes. Thus far, Alexa’s HIPAA-Eligible Skills program allows covered entities to design and launch HIPPA-compliant medical apps that use protected health information; these are available only in the United States [74]. However, the implementation of voice technology into clinical practice requires further regulation. In this context, guidelines and policies for telehealth (ie, those of the American Telemedicine Association) should include rules describing the utilization of medical VAs. This topic is directly associated with security, privacy, and hacking issues [75,76]. Edu et al [77] reviewed a wide range of elements that expose smart speakers at the risk of the attack surface, particularly those associated with the interaction between the user and the smart home personal assistants [77].

**Figure 2:**
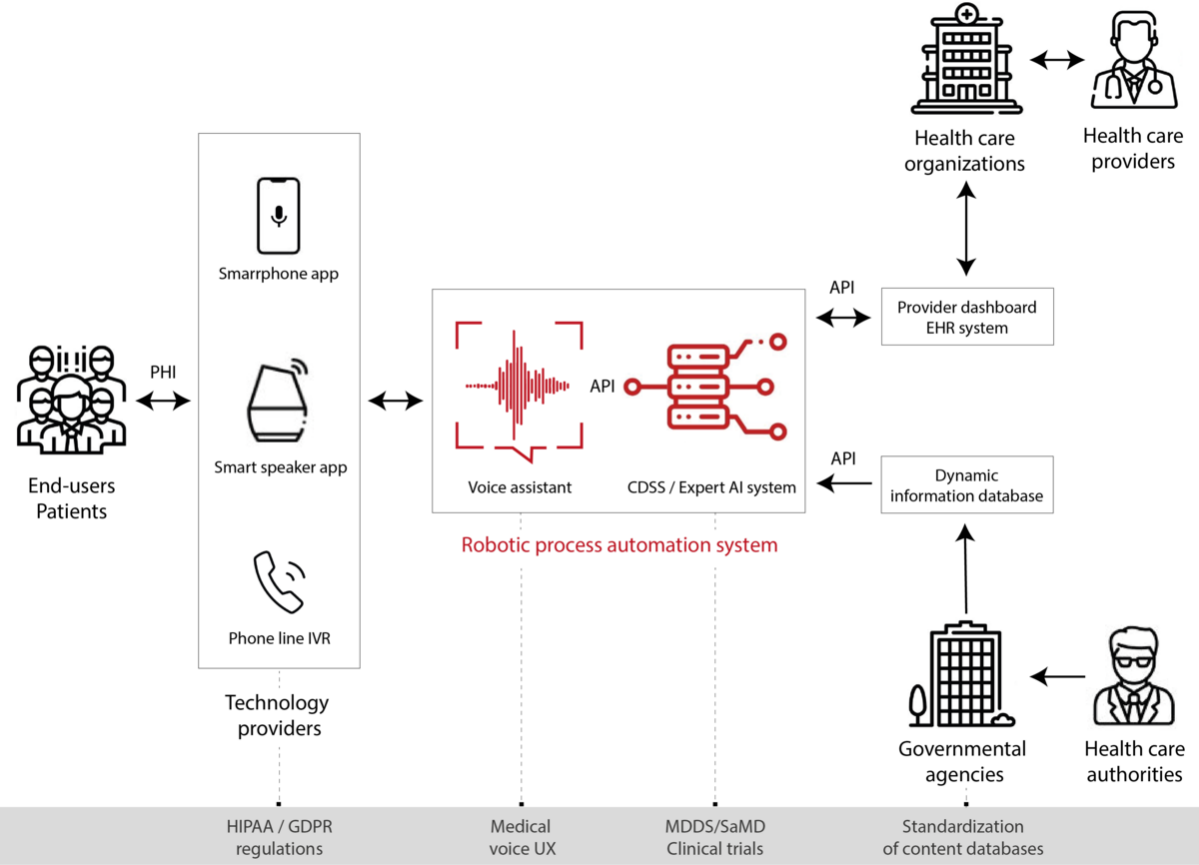
Workflow of the AI-driven voice chatbot in health care delivery. AI: artificial intelligence; API: application programming interface; CDSS: clinical decision support systems; HER: electronic health record; GDPR: General Data Protection Regulation; HIPAA: Health Insurance Portability and Accountability Act of 1996; IVR: interactive voice response; MDDS: Medical Device Data System; PHI: protected health information; SaMD: Software as a Medical Device; UX: user experience.

Amid this rapidly changing landscape, it is important to build relationships amongst the different stakeholders working to implement telemedicine innovations. These stakeholders include retail companies selling the telemedicine technology, software companies interfacing the telemedicine technology with health care systems, health care systems and their providers, payors, patients, and government health agencies (Figure 2).

Close collaboration should focus on the following:

1. Risk-based classification and verification process for VAs including assessment using FDA software as a Medical Device Guidance [15] and Medical Device Data Systems, Medical Image Storage Devices, and Medical Image Communications Devices Guidelines [14].
2. Early clinical validation; that is, using the FDA Early Feasibility Studies Program.
3. Protected health information security including voice patterns and pin-code user verification; currently implemented in HIPAA-eligible Alexa voice apps.
4. Interoperability of the systems to integrate voice-first devices, peripheral devices, and wearables with EHR systems through application programming interfaces; a feasibility study confirmed the integration of the Amazon Alexa-deployed CardioCube app with Centricity (GE Healthcare), OpenEMR, and Epic through an Fast Healthcare Interoperability Resources protocol (Cedars-Sinai Medical Center; under the Cedars-Sinai Accelerator) [78].
5. Harmonization and standardization of medical content databases providing up-to-date information through established data flow channels for quality assurance.
6. Alternative information delivery methods in case of failure in capturing or understanding questions asked by a user. Palanica et al [79] showed that VAs have different comprehension accuracy for drug names from 54.6% to 91.8% for Amazon Alexa and Google Assistant, respectively. Furthermore, Alagha and Helbing [80] showed that VAs have high variability in information quality about vaccines; hence, it is essential to secure a “safe-mode,” redirecting the user to another source of information in case of delivery failure.
7. Segregation and personalization of relevant information in accordance with end-user profiles, taking into consideration determinants including demographics (especially older individuals [81,82] who showed high readiness for voice technology [83]), geographic localization, and spoken languages (voice interfaces are available in a limited number of languages) [84].
8. User experience to optimize how users who seek health information interact with VAs, preferably dedicated medical voice user designers with expertise in health care to understand patients’ needs.
9. Context-aware method of delivery dependent on end-user characteristics and the digital ecosystem; for example, voice-only as a stand-alone smart speaker, smartphone- or computer-deployed app (more applicable for users with broadband internet access), or the telephone (useful for rural areas with limited internet access) vs voice and video devices (ie, Google Nest). In this aspect, VAs might be used for provider-patient audio calls, while devices with embedded video cameras allow audio-visual communication.

Reimbursement for VAs is an important factor determining its adoption. Even though chatbots are not currently eligible for billing both in Europe and in the United States, Keesara et al [50] proposed that the payment structure could be based on time-based models or fixed fee-for-service monetization. Furthermore, the reimbursement for evaluation and management billing codes could be adjusted for digital services, while the Centers for Medicare and Medicaid Services could remove restrictions on in-person consultations under evaluation and management services [50]. Alternatively, having well-established distribution, supply chain, and AI services, software companies developing VAs could become a key player in the health care ecosystem. Interestingly, Amazon Care provides app-based health services to its employees bypassing health plans and brokers. Furthermore, Amazon has enabled patients to order and have their medications delivered home through Amazon Pharmacy. Subsequently, with Amazon’s Pillo Health, an interactive drug dispenser with voice-first technology, patients can optimize pharmacological treatment at home [85]. In the case of health care organizations, Amazon released HealthLake, a HIPAA-eligible data management service functioning as a cloud EHR system [86].

## Future Directions

VAs could be applied to capture human voice utilized as a digital biomarker. The analysis of vocal characteristics is a promising research field that combines AI and clinical medicine. Maor et al [87] reported an independent association between voice signal analysis and hospitalization as well as mortality among patients with heart failure. Furthermore, quantitative voice analysis was shown to be applicable in the diagnosis of neurodegenerative diseases [88] and COVID-19 [89].

## Conclusions

In summary, with the growing demand for telehealth services, VAs could extend the workforce of medical care providers by using AI-powered interfaces to ensure the safety of patients and medical staff. Conversational agents have the potential to become a regular component of health care systems, thereby multiplying medical capacities during the current COVID-19 pandemic and reducing the spread of COVID-19. Moreover, clinical-grade voice AI chatbots can sustainably supplement routine clinical work in the postpandemic period. Voice technology implements synergistic and practical solutions, which have the potential to optimize health care systems and increase preparedness for future COVID-19–like pandemics. Currently, the Amazon Alexa HIPAA-eligible environment enables VAs implemented in the United States to be applied as a professional medical tool.

## Appendix

Multimedia Appendix 1 Video recording presenting CardioCube VA for patients with diabetes.

## Abbreviations

AI: artificial intelligence
CDSS: clinical decision support systems
EHR: electronic health record
FDA: Food and Drug Administration
HIPAA: Health Insurance Portability and Accountability Act of 1996
RPA: robotic process automation
SaMD: Software as a Medical Device
VA: voice assistant
